# 3D N,O-Codoped Egg-Box-Like Carbons with Tuned Channels for High Areal Capacitance Supercapacitors

**DOI:** 10.1007/s40820-020-00416-2

**Published:** 2020-04-01

**Authors:** Feng Wei, Xiaojun He, Lianbo Ma, Hanfang Zhang, Nan Xiao, Jieshan Qiu

**Affiliations:** 1 School of Chemistry and Chemical Engineering, Anhui Key Laboratory of Coal Clean Conversion and High Valued Utilization, Anhui University of Technology, Maanshan, 243002 Anhui People’s Republic of China; 2 School of Materials Science and Engineering, Anhui University of Technology, Maanshan, 243002 Anhui People’s Republic of China; 3 School of Chemical Engineering, Dalian University of Technology, Dalian, 116024 Liaoning People’s Republic of China; 4 College of Chemical Engineering, Beijing University of Chemical Technology, Beijing, 100029 People’s Republic of China

**Keywords:** Egg-box-like carbon, Opened pores in pores, Areal capacitance, All-solid-state supercapacitor

## Abstract

Functional carbonaceous materials for supercapacitors (SCs) without using acid for post-treatment remain a substantial challenge. In this paper, we present a less harmful strategy for preparing three-dimensional (3D) N,O-codoped egg-box-like carbons (EBCs). The as-prepared EBCs with opened pores provide plentiful channels for ion fast transport, ensure the effective contact of EBCs electrodes and electrolytes, and enhance the electron conduction. The nitrogen and oxygen atoms doped in EBCs improve the surface wettability of EBC electrodes and provide the pseudocapacitance. Consequently, the EBCs display a prominent areal capacitance of 39.8 μF cm^−2^ (340 F g^−1^) at 0.106 mA cm^−2^ in 6 M KOH electrolyte. The EBC-based symmetric SC manifests a high areal capacitance to 27.6 μF cm^−2^ (236 F g^−1^) at 0.1075 mA cm^−2^, a good rate capability of 18.8 μF cm^−2^ (160 F g^−1^) at 215 mA cm^−2^ and a long-term cycle stability with only 1.9% decay after 50,000 cycles in aqueous electrolyte. Impressively, even in all-solid-state SC, EBC electrode shows a high areal capacitance of 25.0 μF cm^−2^ (214 F g^−1^) and energy density of 0.0233 mWh cm^−2^. This work provides an acid-free process to prepare electrode materials from industrial by-products for advanced energy storage devices.

**Electronic supplementary material:**

The online version of this article (10.1007/s40820-020-00416-2) contains supplementary material, which is available to authorized users.

## Article Highlights

- A green low-cost route without the acid washing step is used to prepare the N,O-codoped egg-box-like carbons.
- The obtained carbons possess moderate N, O contents and tuned transfer channels with three-dimensional (3D) egg-box-like structures.
- The fabricated electrode exhibits high areal capacitance and long-term cycle stability.

**Electronic supplementary material:**

The online version of this article (10.1007/s40820-020-00416-2) contains supplementary material, which is available to authorized users.

## Graphical abstract

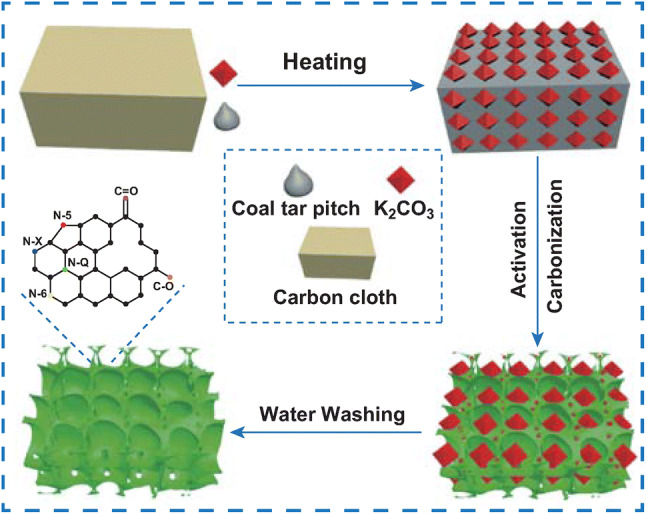

## Introduction

Supercapacitors (SCs) with high power density and rapid charge rates have received extensive attention in the fields of energy storage and conversion [1–4]. A SC is mainly composed of the current collector, electrolyte, and electrode [5]. The properties of SC largely depend on the electrode materials, e.g., carbonaceous materials (CMs) [6, 7], metal oxides [8], conductive polymers [9], and their composites [10–12]. CMs have become the prime choice of electrode materials for SCs due to their excellent electrical conductivity, versatile porosity and high surface area [13–15]. A substantial number of strategies have been reported for the preparation of porous CMs [16–18]. Further, the introduction of heteroatoms in CMs can improve the electrical conductivity, enhance the surface wettability of carbonaceous electrodes, and provide additional pseudocapacitance [19–21]. Nevertheless, the preparation process of CMs is usually complicated and costly. The corrosive chemical reagents not only corrode the equipment but also produce environment pollutants. For instance, *N*-doped porous CMs via SiO_2_ template showed a high areal capacitance of 18.6 μF cm^−2^ [22]. However, the removal of the SiO_2_ template required the use of corrosive HF solution, which creates an environmental issue [23]. Therefore, an acid-free process to synthesize porous CMs for SCs is urgently sought.

Coal tar pitch (CTP), which is a residue fraction from the distillation of coal tar, possesses abundant polycyclic aromatic hydrocarbons, which can be aromatized and polymerized to form diverse cross-linked nanostructures [24, 25]. In this paper, we report a less harmful route to prepare three-dimensional (3D) N,O-codoped egg-box-like carbons (EBCs) from CTP. The as-achieved EBCs feature opened structures with large specific surface areas and many channels for ion transport, which can ensure the effective contact between electrodes and electrolytes. Moreover, EBCs with doped heteroatoms (N and O) can further improve the electrical conductivity and boost the surface wettability of EBCs. The as-prepared EBCs display a high areal capacitance, a satisfactory rate capability and cycle stability. Impressively, even in all-solid-state SCs, the EBC electrode also presents a high areal capacitance and energy density.

## Experimental

### Synthesis of EBCs

CTP was received from Maanshan Iron & Steel Co. Ltd. in China, and other chemicals were received from Aladdin Co. Ltd. In a typical procedure, 2 g CTP and 8 g K_2_CO_3_ were ground and mixed in the solid state. The resultant mixture was dispersed onto the 2 g carbon cloth in the corundum boat in a horizontal tube oven, subsequently heated to *T*  °C (*T* = 750, 800, and 850) with a ramp of 5 °C min^−1^ in flowing ammonia of 10 mL min^−1^ and kept for 1 h at *T*  °C. The obtained samples were washed several times by using distilled water, subsequently dried at 110 °C for 24 h. The final products were denominated as EBC_750_, EBC_800_, and EBC_850_ when the heat treatment temperature (*T*) was set at 750, 800, and 850 °C, respectively.

### Characterization

The EBC materials were characterized by field emission scanning electron microscopy (FESEM,), transmission electron microscopy (TEM), X-ray photoelectron spectroscopy (XPS), Fourier transform infrared spectrometer (FTIR), X-ray diffraction (XRD), Raman spectroscopy, and a nitrogen adsorption/desorption technique. The elemental analysis was performed using the elemental analyzer. The conductivities of the EBCs were measured by a four-probe method using a source measure unit. The contact angle of EBC electrodes was carried out by the OCA15Pro contact angle tester. Please refer to the Supplementary File for details.

### Electrochemical Test of EBC Electrodes

EBC (90 wt%) and polytetrafluoroethylene (10 wt%) were blended in deionized water without the addition of a conductive agent to make the slurry, subsequently rolled into film and cut into round films (12 mm in diameter), and then dried at 110 °C for 2 h. The round films were pressed onto nickel foam at 20 MPa, followed by immersion in 6 M KOH aqueous electrolyte in vacuum. A coin-type SC was fabricated with two immersed round films and separated by polyethylene membrane. The areal loading of active material on each EBC electrode is ca. 2.15 mg cm^−2^. The total mass of the current collector and active material is ca. 32 mg.

The fabrication of all-solid-state SC is described as follows: Polymeric gel electrolyte of KOH/polyvinyl alcohol (PVA) was prepared according to the reported method [26], which involved stirring 25 mL deionized water, 1 g KOH and 1 g PVA at 95 °C for 2 h. The EBC_800_ electrodes were soaked in KOH/PVA electrolyte for 1 min and then dried in room temperature conditions for 24 h. The all-solid-state SC was assembled with two similar soaked EBC_800_ electrodes. The areal loading of active material on EBC_800_ electrode is ca. 2.18 mg cm^−2^. For the electrochemical measurements of SCs and the calculation methods for gravimetric capacitance, areal capacitance, coulumbic efficiency, energy density and average power density, please refer to the Supplementary File.

## Results and Discussion

### Structure Characterization

**Fig. 1:**
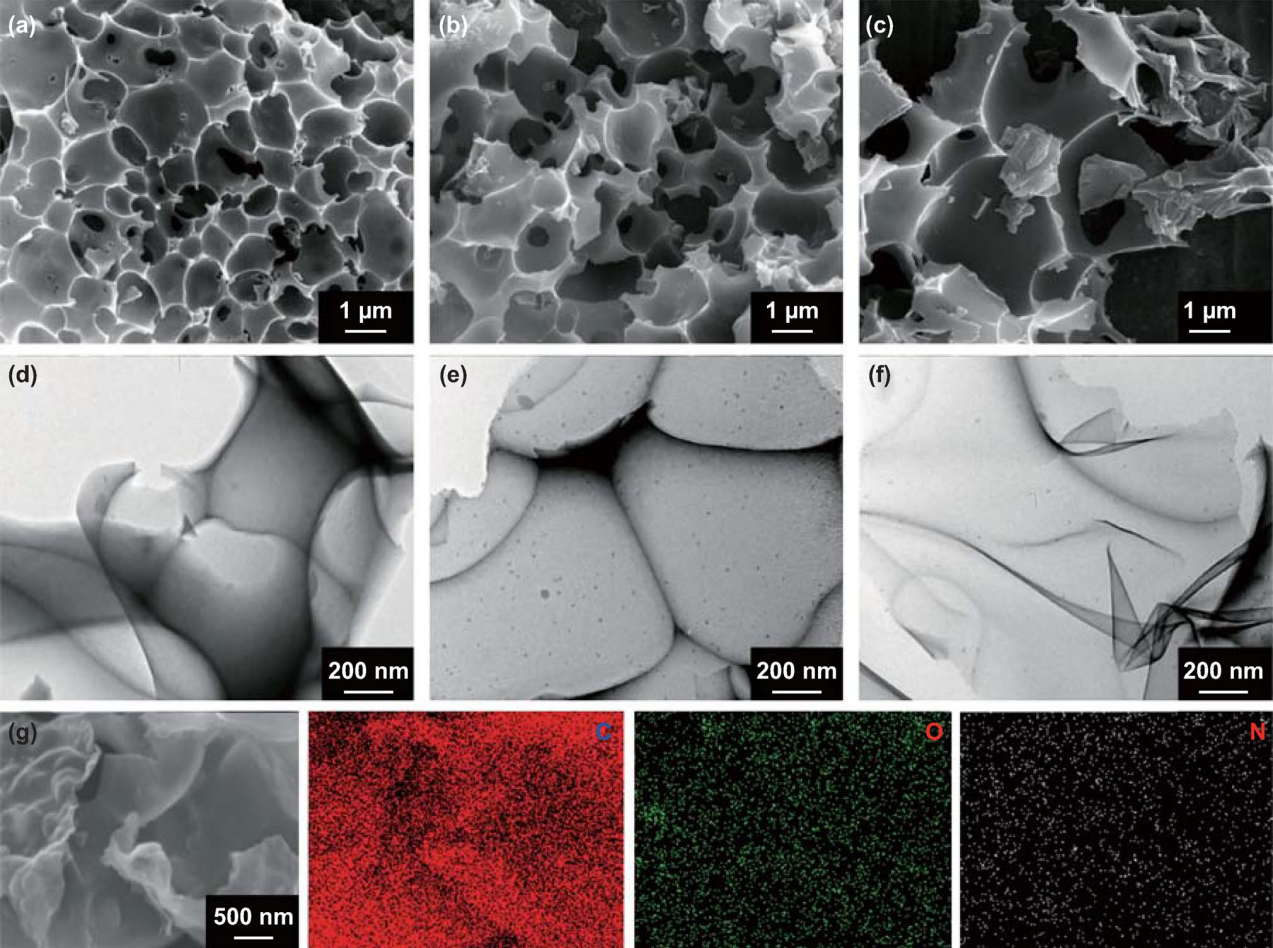
FESEM images of **a** EBC_750_, **b** EBC_800_, and **c** EBC_850_. TEM images of **d** EBC_750_, **e** EBC_800_, and **f** EBC_850_. **g** FESEM image and corresponding EDS mappings of EBC_800_

The FESEM images in Fig. 1a–c show 3D interconnected egg-box-like structures with opened pores in the pores of the as-prepared EBCs. The size of the opened pores in EBC_750_ is ca. 0.4–2 μm (Fig. 1a). The size of the pores in Fig. 1b is larger than that in Fig. 1a due to the tailor/activation function of K_2_CO_3_ as the temperature increases. As expected, some egg-box-like structures are broken due to the highest heat treatment temperature (Fig. 1c). The TEM images in Fig. 1d–f present 3D interconnected porous structures of EBCs. Notably, the pore size in EBC_800_ (Fig. 1e) is larger than that in EBC_750_ (Fig. 1d). EBC_850_ exhibits ultrathin architectures due to the tailor function of K_2_CO_3_ at higher temperatures (Fig. 1f). The HRTEM image of EBC_800_ shows that EBC_800_ features local graphitization with a thickness of only ca. 6.5 nm (Fig. S1). The energy-dispersive spectrometer (EDS) mapping images of EBC_800_ (Fig. 1g) display a relatively homogeneous distribution of C, N, and O elements, which confirm that N and O atoms were successfully doped in EBCs.

**Fig. 2:**
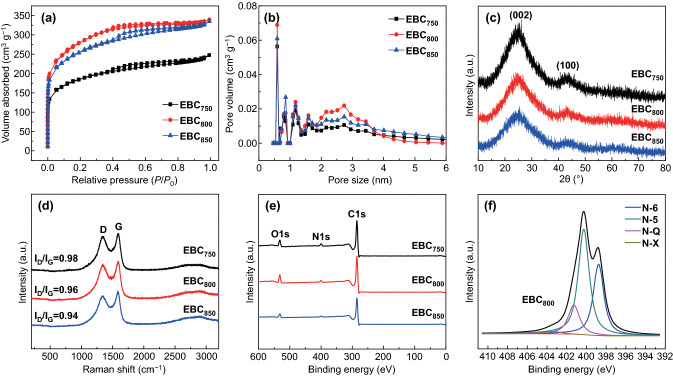
**a** Nitrogen adsorption/desorption isotherms, **b** pore size distribution curves, **c** XRD patterns, **d** Raman spectra, **e** survey XPS spectra of EBCs, **f** high-resolution XPS spectrum at N 1s region of EBC_800_

The nitrogen adsorption/desorption isotherms of three EBC samples are shown in Fig. 2a. All of the isotherms display hierarchical pore structures, e.g., typical IV-type curves with strong adsorption at *P*/*P*_0_ < 0.01 and small hysteresis loops at 0.4 < *P*/*P*_0_ < 0.95, which indicates the coexistence of micropores and mesopores. The micropores can be functioned as ion-adsorption active sites, while mesopores provide channels for ion fast transport [27]. The pore size of most of the EBC samples is less than 3 nm due to the in situ activation role of K_2_CO_3_ (Fig. 2b). *S*_BET_ of EBCs increases from 604 to 854 m^2^ g^−1^ and then decreases to 834 m^2^ g^−1^ when the heat treatment temperature increases from 750 to 850 °C, while *D*_ap_ of the EBC samples increases from 2.27 to 2.43 nm (Table 1). The yields of EBC_750_, EBC_800_ and EBC_850_ are 34.27%, 22.78%, and 16.55%, respectively. The foregoing results suggest that the pore structure parameters and yields of EBCs are easily tuned by changing the heat treatment temperatures.

**Table 1:** Pore structure parameters and yields of EBCs

Samples	*D*_ap_ (nm)	*S*_BET_ (m^2^ g^−1^)	*V*_t_ (cm^3^ g^−1^)	*V*_mic_ (cm^3^ g^−1^)	*V*_mic_/*V*_t_ (%)	Yield (%)
EBC_750_	2.27	604	0.37	0.14	37.84	34.27
EBC_800_	2.41	854	0.48	0.21	43.75	22.78
EBC_850_	2.43	834	0.51	0.18	35.29	16.55

*D*_ap_ average pore diameter, *S*_BET_ specific surface area, *V*_t_ total pore volume, *V*_mic_ micropore volume

The XRD patterns of EBCs only present the (002) and (100) peaks at ca. 25° and 43° (Fig. 2c). The *d*_002_ decreases from 0.3576 nm for EBC_750_ to 0.3515 nm for EBC_850_, while *L*_c_ increases from 1.1574 to 1.2417 nm, which reveals that the graphitization degree and crystallite height increases with an increase in the heat treatment temperature from 750 to 850 °C (Table S1). The Raman spectra display a typical D-band and G-band of carbonaceous materials (Fig. 2d). The D-band located at ca. 1344 cm^−1^ is related to the defects and disorder structures [28], while the G-band at ca. 1592 cm^−1^ can be assigned to the graphitic structures [29]. The peak intensity ratio of the D-band to G-band (*I*_D_/*I*_G_) of EBC_850_ (0.94) is lower than that of EBC_750_ (0.98) and EBC_800_ (0.96), which suggests that EBC_850_ possesses the highest graphitization degree among the three samples, which is consistent with the XRD results. The electrical conductivity of EBC_800_ is 508 S m^−1^, which is higher than that of EBC_750_ (84 S m^−1^) and EBC_850_ (242 S m^−1^), and higher than that of the interconnected carbon nanosheet (226 S m^−1^) [30]. The survey XPS spectra (Fig. 2e) of the three samples only present two strong peaks at 284.9 and 532.4 eV and present a weak peak at 399.8 eV, which corresponds to the peaks of C 1*s*, O 1*s*, and N 1*s*, respectively. The XRD and XPS results indicate that the impurities can be removed by using water washing, that is, this synthesis route avoids the acid washing step, simplifies the preparation process and reduces the cost. The O 1*s* spectra of the EBC samples were deconvoluted into two peaks: C=O (532.1 eV) and C–O (533.3 eV) (Fig. S2a–c) [31]. The N 1*s* spectra of EBCs can be fitted into four peaks, which correspond to pyridinic N (N-6, 398.8 eV), pyrrolic N (N-5, 400.3 eV), quaternary N (N-Q, 401.3 eV), and oxidized pyridinic N (N-X, 403.4 eV) groups (Figs. 2f and S2d, e) [32]. The maximum O and N contents in EBC_800_ are 8.21 and 3.55 at%, respectively (Table S2), which shows agreement with the element analysis results (Table S3). The N-5 and N-6 contributed to the pseudocapacitive interactions, which improved the capacitance. The N-Q and N-X can effectively facilitate electron transfer, which enhances the conductivity of carbon materials [6]. Figure S3 exhibits the FTIR spectra of the EBC samples. All of the EBC samples present absorption peaks at ca. 1080, 1387, and 1625 cm^−1^, which correspond to stretching vibrations of C=O, C–N, and C–O [33], respectively, which is consistent with the results of the XPS analysis. The water contact angle of EBC_750_, EBC_800_, and EBC_850_ is 19.1°, 18.6°, and 39.4°, respectively (Fig. S4), which is obviously lower than that of porous carbon nanosheets (84°–127°), indicates that the residual O functionalities greatly improve the wettability of EBCs [14].

**Fig. 3:**
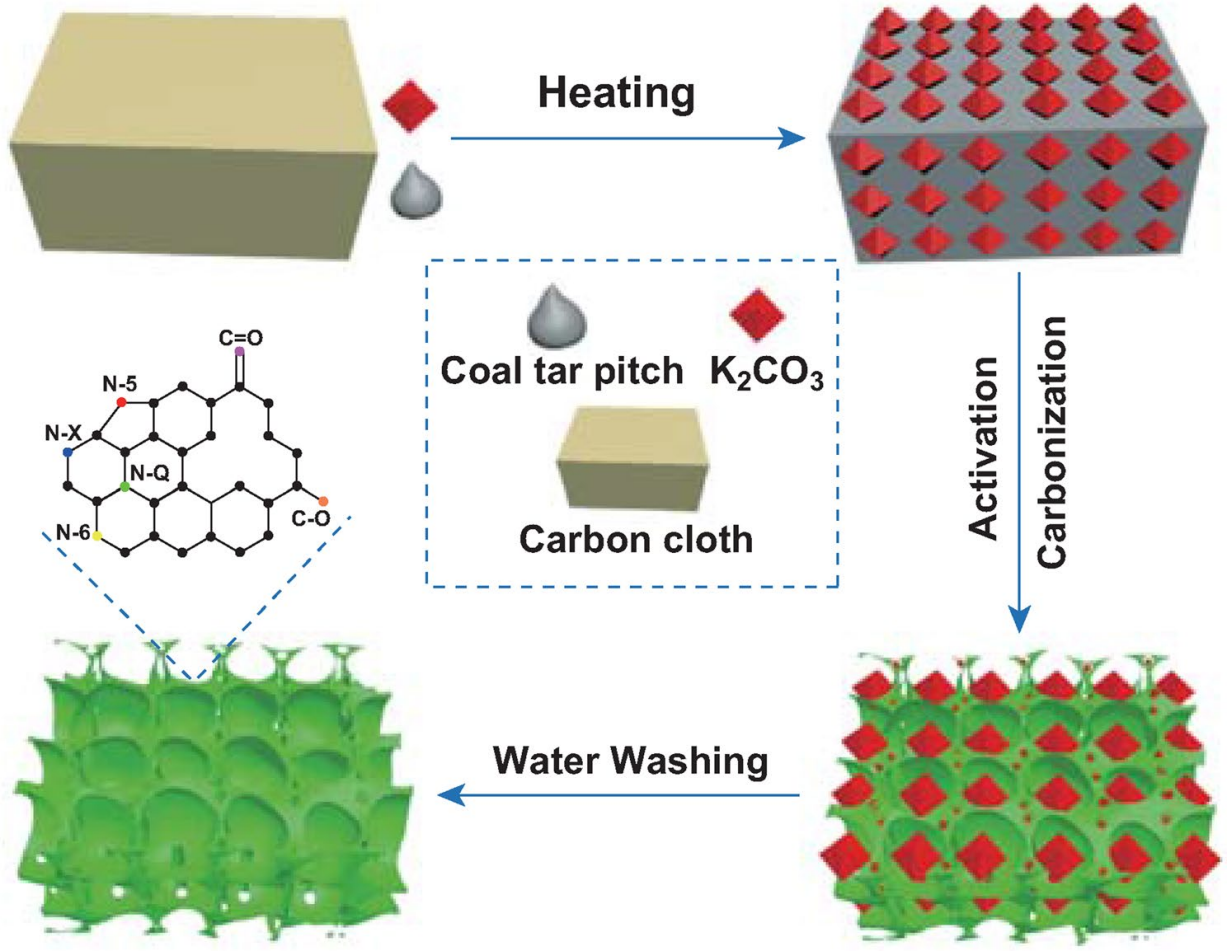
Synthetic scheme of EBCs from CTP using carbon cloth as the directing template combined with in situ K_2_CO_3_ activation

The synthetic scheme of EBCs from CTP is shown in Fig. 3. CTP and K_2_CO_3_ were mixed in the solid state and then distributed on the surface of the carbon cloth. Subsequently, during the heating process, the liquefied CTP diffused and coated onto the surfaces of the K_2_CO_3_ particles and carbon cloth to form 3D interconnected films. As the heat treatment temperature gradually increased to 600 °C, K_2_CO_3_ started to react with carbon as follows: K_2_CO_3_ + 2C → 2K + 3CO [28]. Carbon cloth served as a template to direct the transformation of aromatic hydrocarbon molecules in the CTP into EBCs. Benefiting from the synergetic effect of the in situ tailor/activation of K_2_CO_3_ and the directing function of the carbon cloth, EBCs with hierarchical pores and egg-box-like structures are generated simultaneously. By simply washing with deionized water, the final product of 3D N,O-codoped EBCs with opened pores in pores is achieved.

**Fig. 4:**
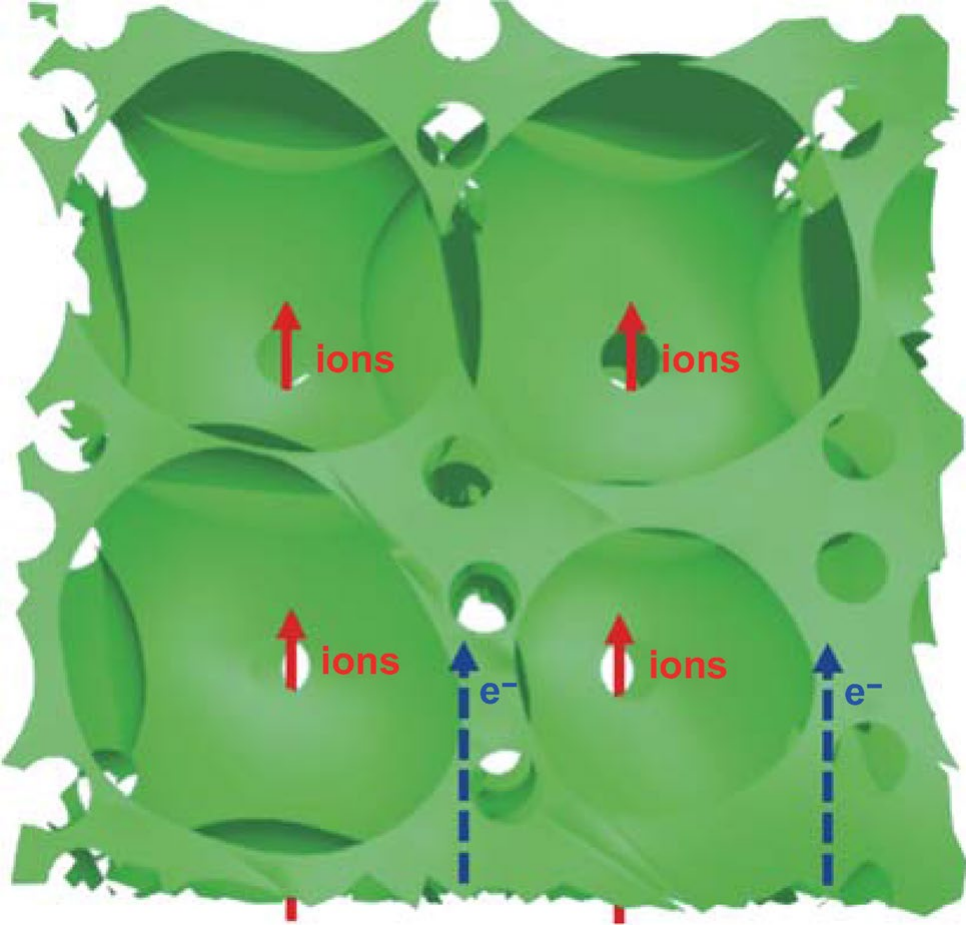
Schematic of ion transport and electron conduction in 3D EBC electrodes

Figure 4 shows the schematic of the ion transport and electron conduction in 3D EBC electrodes. The 3D interconnected structures offer highways for electron conduction, which enables superior cycle stability. On the other hand, the pore-in-pore structures provide short channels for fast ion transport, which yields a satisfactory rate performance and high areal capacitance. According to formula [34],

$$\tau_{0} = \frac{{L^{2} }}{qD},$$

where *τ*_0_, *q*, *D,* and *L* are the time constants, a dimensionality-dependent constant (*q *= 2, 4, or 6 for one-dimensional diffusion, two-dimensional diffusion, or three-dimensional diffusion, respectively), diffusion coefficient, diffusion distance, respectively, the ion diffusion time in 3D EBC electrodes is shorter than that in 1D and 2D materials, which enables a better rate capability.

### Electrochemical Performance

**Fig. 5:**
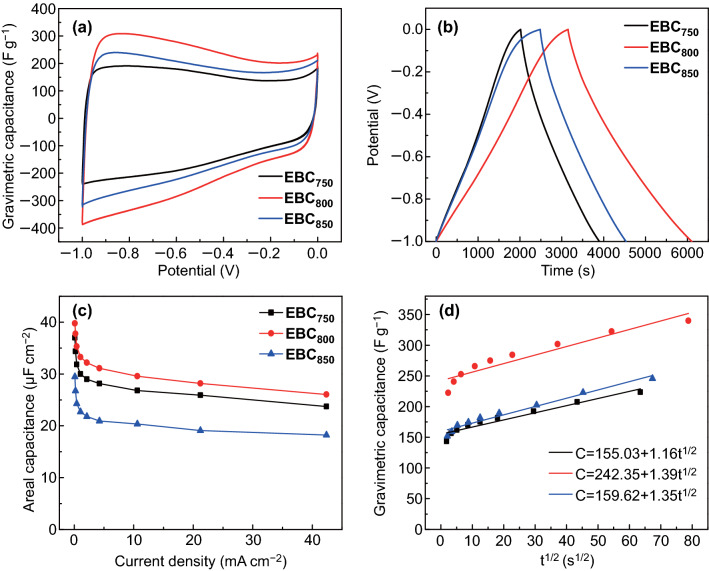
Electrochemical performance of EBC electrodes in a three-electrode system in 6 M KOH electrolyte: **a** CV profiles at 5 mV s^−1^, **b** GCD curves at 0.212 mA cm^−2^, **c** areal capacitance at various current densities, and **d** gravimetric capacitance versus *t*^1/2^

Benefiting from the 3D egg-box-like structures with opened pores in pores, high mesopore content, and moderate N, O doping level, the as-obtained EBC samples are expected to be ideal electrode materials for SCs. The electrochemical performance of the EBC samples was tested by cyclic voltammetry (CV) and galvanostatic charge–discharge (GCD) measurements using a three-electrode system in 6 M KOH electrolyte. For the Hg/HgO electrode, platinum foil was employed as the reference and counter electrode, respectively. The areal loading of active material on each EBC electrode is ca. 2.12 mg cm^−2^. At the scan rate of 5 mV s^−1^, the CV curves (Fig. 5a) of the EBC electrodes exhibit a quasi-rectangular shape with redox humps. The GCD curves (Fig. 5b) of the EBC electrodes at 0.212 mA cm^−2^ show linear shapes with a slight deviation from the line. The deviation of both the CV and GCD curves reveal the presence of pseudocapacitance caused by the faradaic reaction of the N and O functionalities. The areal capacitance is 37.0 μF cm^−2^ (223 F g^−1^), 39.8 μF cm^−2^ (340 F g^−1^), and 29.5 μF cm^−2^ (246 F g^−1^) for EBC_750_, EBC_800_, and EBC_850_, respectively, at the current density of 0.106 mA cm^−2^ (Fig. 5c). The EBC800 electrode achieves a high areal capacitance to 29.6 μF cm^−2^ (253 F g^−1^) at 10.6 mA cm^−2^ and maintains 26.0 μF cm^−2^ (222 F g^−1^) at 42.4 mA cm^−2^.

The electrochemical kinetic of EBCs was investigated to understand the nature of the charge storage mechanism and contribution of N,O-containing functional groups on pseudocapacitance. Generally, the capacitance (*C*) can be calculated via the following equation: $C = k_{1} + k_{2} t^{1/2}$, where *k*_1_, *k*_2_*t*^1/2^, *t* represents the surface capacitive effects (*C*_E_, related to the EDLC), diffusion controlled process (*C*_P_, ascribed to pseudocapacitance) and discharge time, respectively [35]. The *k*_1_ and *k*_2_ for EBC_750_ are 155.03 and 1.16, respectively, while that for EBC_800_ is 242.35 and 1.39, respectively, and that for EBC_850_ is 159.62 and 1.35, respectively (Fig. 5d). For EBC_800_, the contribution of *C*_P_ on *C* decreases as the current density increases. The *C*_E_ and *C*_P_ values of EBC_800_ electrode are higher than that of EBC_750_ and EBC_850_ electrodes at the same current density. At 0.106 mA cm^−2^, among the three EBC electrodes, EBC_800_ presents the highest *C*_E_ of 242.4 F g^−1^ due to its largest *S*_BET_ and presents the highest *C*_P_ of 97.3 F g^−1^ due to its highest total content of O and N heteroatom (11.76 at %), as shown in Table S2.

**Fig. 6:**
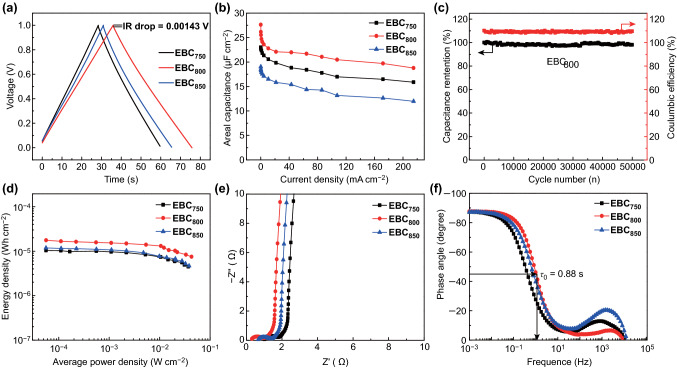
**a** GCD curves of EBC electrodes at 1 A g^−1^. **b** Areal capacitance of EBC electrodes at various current densities. **c** Capacitance retention and coulombic efficiency of EBC_800_-based SC after 50,000 cycles at 10.75 mA cm^−2^. **d** Ragone plots of EBC capacitors. **e** Nyquist plots of EBC electrodes. **f** Bode plots of phase angle versus frequency

The electrochemical performance of EBC electrodes is evaluated in the symmetric coin-type SCs in 6 M KOH electrolyte. The GCD curves of EBCs present symmetrical shapes (Fig. 6a), which suggest the ideal electric double layer capacitor (EDLC) behavior. The IR drop of EBC_750_, EBC_800_, and EBC_850_ is only 0.00262, 0.00143, and 0.00257 V, respectively, which confirms the excellent electric conductivity of EBCs [36]. At the current density of 0.1075 mA cm^−2^, the areal capacitance of EBC_750_, EBC_800_ and EBC_850_ is 23.0 μF cm^−2^ (139 F g^−1^), 27.6 μF cm^−2^ (236 F g^−1^) and 19.0 μF cm^−2^ (159 F g^−1^), respectively (Figs. 6b and S5). Even at a very high current density of 215 mA cm^−2^, the areal capacitance of EBC_800_ can be retained at 18.8 μF cm^−2^ (160 F g^−1^), while for EBC_750_ and EBC_850_, the areal capacitance is 12.0 and 15.8 μF cm^−2^, respectively. Although the difference between EBC_800_ and EBC_850_ in S_BET_ is small, the areal capacitance of EBC_800_ is higher than that of EBC_850_, which is due to the higher content of N, O heteroatom. The gravimetric and areal capacitance of EBC_800_ at the current density of 0.1075–215 mA cm^−2^ is the highest due to the largest *S*_BET_ of EBC_800_ and the optimal O content among the three samples. The areal capacitance of EBC_800_ is also higher than those of carbon-based electrodes reported in the literature (as summarized in Table 2) [37–48]. The high capacitance and excellent rate capability of EBCs are ascribed to its 3D interconnected egg-box-like structures with opened pores in pores, which provide plentiful channels for ion fast transport, abundant active sites for ion adsorption and highways for electron conduction [49]. Moreover, the oxygen-containing groups are conducive to boosting the surface wettability of the EBC electrodes in KOH electrolyte. Furthermore, the moderate N-containing functional groups are beneficial to provide pseudocapacitance, which causes high capacitance. A long cycle life is another key factor for the practical application of SCs. The maximum capacitance retention of EBC_800_-based SC is 98.1% even after 50,000 cycles at 10.75 mA cm^−2^ (Fig. 6c), which exhibits a long-term cycle stability. Additionally, the EBC_800_-based SC displays a high coulombic efficiency of 109.7% after 50,000 cycles, which implies excellent reversibility. This finding proves that the EBC_800_-based SC can be used as a long-life energy storage device. In addition, the energy density of the EBC_800_ capacitor is 0.01763 mWh cm^−2^ at the power density of 0.056 mW cm^−2^ (Fig. 6d), which confirms its potential application.

**Table 2:** Comparison of the gravimetric and areal capacitance of EBC_800_ electrode with reported carbon-based electrodes

Materials	*S*_BET_ (m^2^ g^−1^)	Electrolyte	Current density (A g^−1^)	*C*_g_ (F g^−1^)	*C*_a_ (μF cm^−2^)	Refs.
EBC_800_	854	6 M KOH	0.05	236	27.6	This work
			100	160	18.8	
BFC	1350	6 M KOH	1.0	200	14.8	[37]
CCNC1	2561	6 M KOH	0.1	205	8.0	[38]
SRPC-4 K-900	2143	6 M KOH	0.5	276	12.9	[39]
2D-HPC	2406	6 M KOH	0.5	280	11.6	[40]
PCG	1615	6 M KOH	0.5	220	13.6	[41]
3DG1000	1545	6 M KOH	1.0	231	15.0	[42]
HPCNT	1419	6 M KOH	0.1	248	17.5	[43]
IMPC	1327	1 M H_2_SO_4_	0.5	254	19.1	[44]
HP-CF	1175	6 M KOH	0.5	238	20.3	[45]
UCM-1	1267	6 M KOH	0.5	227	17.9	[46]
FGH-150	1006	6 M KOH	0.2	227	22.6	[47]
HTYC-2:1	1300	6 M KOH	2.0	225	17.3	[48]

The CV curves of EBC electrodes show a rectangular shape at the scan rate of 2 mV s^−1^ (Fig. S6a–c). No obvious distortions in the CV curves are observed when the scan rate increases to 200 mV s^−1^, which exhibits the ion fast transport within electrodes and satisfactory rate capability (Fig. S6d). The Nyquist plots of EBC electrodes are composed of a straight line in the low frequency region and a semicircle in the high-frequency region (Fig. 6e), and the corresponding electric equivalent circuit model is displayed in Fig. S7. The straight line in the low frequency region exhibits ideal capacitive behavior [21]. The x-intercept of the Z′ axis corresponds to the intrinsic ohmic resistance (*R*_s_) of EBC electrodes, while the diameter of the semicircle represents the charge transfer resistance (*R*_ct_) [50]. The short x-intercept and small diameter of a semicircle prove that the EBC_800_ electrode has low internal resistance and charge transfer resistance due to its novel 3D interconnected N,O-codoped egg-box-like structures with opened pores in pores to provide channels for ion transport and electron conduction. In the Bode plots, the phase angle of EBC_750_, EBC_800_ and EBC_850_ is − 87.2°, − 87.6°, and − 87.2°, respectively, at 0.001 Hz, which indicates the excellent EDLC behavior [51]. The characteristic frequency (*f*_0_) at the phase angle of − 45° for EBC_750_, EBC_800_, and EBC_850_ is 0.42, 1.14, and 0.76 Hz, respectively, which correspond to the relaxation time (*τ*_0_, *τ*_0_ = 1/*f*_0_) of only 2.38, 0.88, and 1.32 s, respectively (Fig. 6f), which confirms the best charge/discharge rate of EBC_800_ among the three EBC samples [52, 53].

The all-solid-state SC with a symmetric two-electrode configuration was also fabricated based on the EBC_800_ electrode and KOH/PVA gel polymer electrolyte to further ascertain the electrochemical performance of the EBC samples (Fig. S8). The CV curves of EBC_800_ at various scan rates exhibit a quasi-rectangular shape, which suggests a satisfactory capacitive behavior (Fig. S8a). The GCD curve of EBC_800_ presents an approximately symmetric triangular-shape at the current density of 0.218 mA cm^−2^ (Fig. S8b). At the current density of 0.109 mA cm^−2^, the areal capacitance of EBC_800_ is 25.0 μF cm^−2^, which corresponds to the gravimetric capacitance of 214 F g^−1^, and the areal capacitance of EBC_800_ remains 15.2 μF cm^−2^ (130 F g^−1^) at 43.6 mA cm^−2^ with a capacitance retention of 60.5% (Fig. S8c). The gravimetric capacitance of the all-solid-state SC based on EBC_800_ electrode is higher than those of the reported carbonaceous electrodes, e.g., hierarchically graphene nanocomposite (180 F g^−1^) [54], activated carbon/carbon nanotube/reduced graphene oxide film (101 F g^−1^) [33], waste sugar-based carbon materials (105 F g^−1^) [55], and 3D graphene hydrogel film (186 F g^−1^) [56]. The capacitance retention of the EBC_800_-based all-solid-state SC is 96.1% after 10,000 cycles at 10.9 mA cm^−2^, which also display excellent cycle stability (Fig. S8d). In addition, the EBC_800_-based all-solid-state SC presents a high coulombic efficiency of 98.5% after 10,000 cycles. The EBC_800_ capacitor shows a high energy density of 0.0233 mWh cm^−2^ at the power density of 0.0979 mW cm^−2^ (Fig. S8e). The *R*_s_ of EBC_800_ electrode is only 1.35 Ohm, as shown in Fig. S8f. Moreover, the negligible semicircle in the high-frequency region also proves a low *R*_ct_ of EBC_800_ sample. EBCs hold a potential application in SCs, as evidenced by their high areal capacitance, excellent rate capability and long-term cycle stability in aqueous and all-solid-state SCs. The satisfactory electrochemical performance of the EBC_800_ electrode is explained as follows: (1) the pore-in-pore structures offer short channels for fast ion transport, which yields excellent rate performance and high areal capacitance; (2) the 3D interconnected networks provide highways for electron conduction, which produces superior cycle stability; (3) the oxygen-containing groups are conducive to boosting the surface wettability of the EBC_800_ electrode in KOH electrolyte; (4) the N, O functionalities are beneficial for providing pseudocapacitance, which delivers a high capacitance.

## Conclusion

For the first time, a less harmful route was reported in this paper to prepare 3D N,O-codoped EBCs from CTP for SC application. This route avoids the acid washing step, simplifies the process and reduces the cost. Benefiting from the 3D interconnected egg-box-like structures with opened pores in pores and N,O-containing functional groups, the EBCs exhibit a prominent areal capacitance of 39.8 μF cm^−2^ at 0.106 mA cm^−2^ in aqueous electrolyte. Furthermore, as an electrode for SC, EBC presents a high areal capacitance of 27.6 μF cm^−2^ at 0.1075 mA cm^−2^ and a long-term cycle stability with only 1.9% decay after 50,000 cycles. In addition, the EBC electrode shows a high areal capacitance of 25.0 μF cm^−2^ and an energy density of 0.0233 mWh cm^−2^ even in all-solid-state SCs. This work provides an acid-free process for the synthesis of carbon-based electrode materials from industrial residual pitch-based carbon sources for long-life energy storage devices.

## Electronic supplementary material

Below is the link to the electronic supplementary material.

Supplementary material 1 (PDF 700 kb)
