# Supplementary material for: 3D N,O-Codoped Egg-Box-Like Carbons with Tuned Channels for High Areal Capacitance Supercapacitors
Source: Nanomicro Lett. 2020 Apr 1;12:82. doi: 10.1007/s40820-020-00416-2 (PMC7770960; doi:10.1007/s40820-020-00416-2)
Supplement: Supplementary file 1 — Supplementary material 1 (PDF 700 kb) [file 40820_2020_416_MOESM1_ESM.pdf]

Supporting Information for

3D N, O-codoped Egg-box-like Carbons with Tuned Channels for High Areal Capacitance Supercapacitors

Feng Wei[1], Xiaojun He[1, *], Lianbo Ma[2], Hanfang Zhang[1], Nan Xiao[3], Jieshan Qiu[4, *]

[1]School of Chemistry and Chemical Engineering, Anhui Key Laboratory of Coal Clean Conversion and High Valued Utilization, Anhui University of Technology, Maanshan 243002, Anhui, People's Republic of China

[2]School of Materials Science and Engineering, Anhui University of Technology, Maanshan 243002, Anhui, People's Republic of China

[3]School of Chemical Engineering, Dalian University of Technology, Dalian 116024, Shandong, People's Republic of China

[4]College of Chemical Engineering, Beijing University of Chemical Technology, Beijing 100029, People's Republic of China

*Corresponding authors. E-mail: agdxjhe@126.com (X.J. He), qiujs@mail.buct.edu.cn (J.S. Qiu)

S1 Experimental Section

Characterization: The EBC materials were characterized by field emission scanning electron microscopy (FESEM, Nano-430, USA), transmission electron microscopy (TEM, JEM-2100, Japan), X-ray photoelectron spectroscopy (XPS, Thermo EBCALAB250, USA), Fourier transform infrared spectrometer (FTIR, Nicolet6700, USA), X-ray diffraction (XRD, D8ADVANCE, German) and Raman spectroscopy (INVIA, England). The pore structure of EBCs was tested by nitrogen adsorption/desorption technique at -196 °C (Micrometrics, ASAP2460, USA), and the specific surface area of EBCs was calculated by the Brunauer-Emmett-Teller (BET) method in the relative pressure ranges from 0.05 to 0.30. The elemental analysis was performed on the elemental analyzer (Vario EL III). The conductivity of EBCs was measured by a four-probe method using a source measure unit (Keithley 6430). The contact angle of EBC electrodes was carried out by OCA15Pro contact angle tester (DataPhysics, Germany).

The average carbon crystallite lattice parameters: inter-layer spacing (d_{002}) and crystallite height (L_c) were determined on basis of XRD results *via* the Bragg [S1] and Scherrer equations [S2] (Eqs. S1 and S2):

$$2d_{002} sin\theta = \lambda \qquad \text{(S1)}$$

$$L_c = \frac{0.89\lambda}{B_c cos\theta_c} \quad \text{(S2)}$$

Where λ (nm), θ_c (°), B_c (°) stands for the X-ray wavelength (0.154056 nm for copper Kα radiation), the peak position of (002) band and the full width at half maximum (FWHM) of the corresponding band, respectively.

Electrochemical Test of EBC Electrodes: The supercapacitive properties were evaluated by cyclic voltammetry (CV) on a CHI760E electrochemical workstation (Chenhua Instrument, China). The galvanostatic charge-discharge (GCD) was performed on the supercapacitor test system (SCTS, Arbin Instruments, USA). The electrochemical impedance spectroscopy (EIS) measurement was performed by using a SI 1260 Solartron impedance analyzer (Solartron Analytical, UK) with a SI 1287 Solartron potentiostat.

For the three-electrode cell and the symmetric two-electrode cell, the gravimetric capacitance (C_g, F g^{-1}) of EBC electrodes was calculated from the GCD curves by the Eqs. S3 and S4, respectively.

$$C_g = \frac{I}{m\frac{\Delta V}{\Delta t}} \quad \text{(S3)}$$

$$C_g = \frac{4I}{m\frac{\Delta V}{\Delta t}} \quad \text{(S4)}$$

Where I (A), ΔV (V), Δt (s) and m (g) signifies the discharge current, the discharge voltage after the IR drop, the discharge time and total mass of active materials (EBCs) in both electrodes, respectively.

The coulumbic efficiency (η) of EBC-based SC was calculated *via* Eq. S5.

$$\eta = \frac{t_d}{t_c} \quad \text{(S5)}$$

Where t_d (s), t_c (s) is the discharge and charge time, respectively.

The areal capacitance (C_a, μF cm^{-2}) of EBC electrodes was obtained *via* Eq. S6:

$$C_a = \frac{C_g}{S_{BET}} \quad \text{(S6)}$$

The energy density (E_g, Wh kg^{-1}) and average power density (P_g, W kg^{-1}) of EBC electrodes were calculated *via* Eqs. S7 and S8, respectively.

$$E_g = \frac{1}{2\times4\times3.6}CV^2 \quad \text{(S7)}$$

$$P_g = \frac{E_g}{\Delta t} \tag{S8}$$

Where V (V), Δt (h) stands for the usable voltage after the IR drop and the discharge time, respectively.

The areal energy density (E_A, Wh cm^{-2}) and power density (P_A, W cm^{-2}) of EBC electrodes were calculated *via* Eqs. S9 and S10, respectively.

$$E_A = \frac{E_g \times m_A}{10^6} \tag{S9}$$

$$P_A = \frac{P_g \times m_A}{10^6} \tag{S10}$$

Where m_A (mg cm^{-2}) is the areal loading of active material on each EBC electrode

S2 Supplementary Figures and Tables

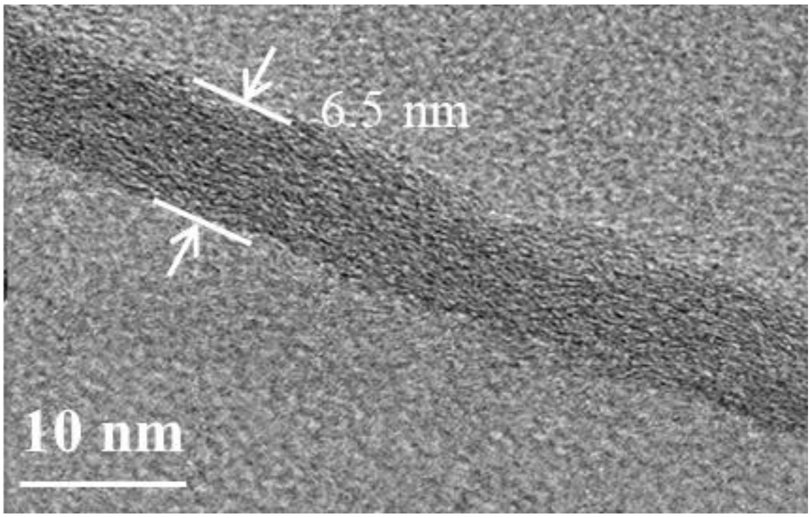

Fig. S1 HRTEM image of EBC_{800}

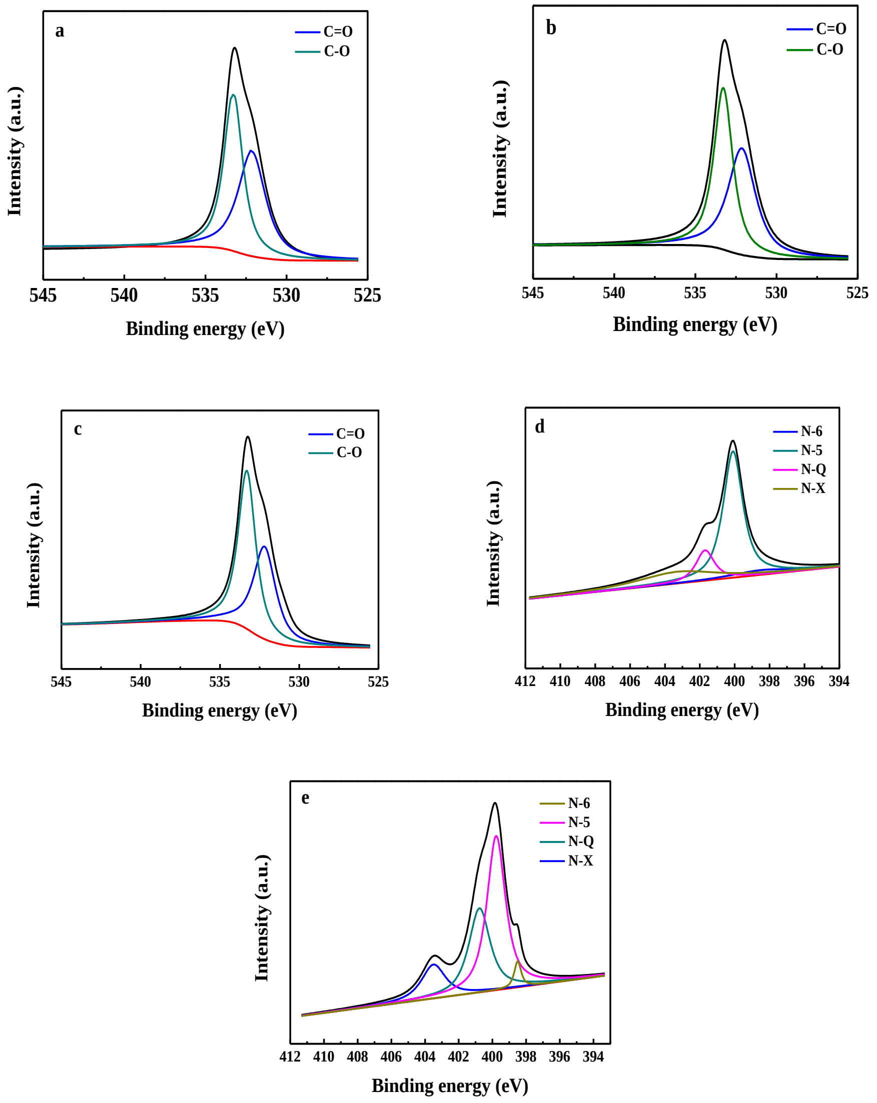

Fig. S2 High-resolution XPS spectra at O1s region of (**a**) EBC_{750}, (**b**) EBC_{800}, (**c**) EBC_{850}; high-resolution XPS spectra at N1s region of (**d**) EBC_{750}, (**e**) EBC_{850}

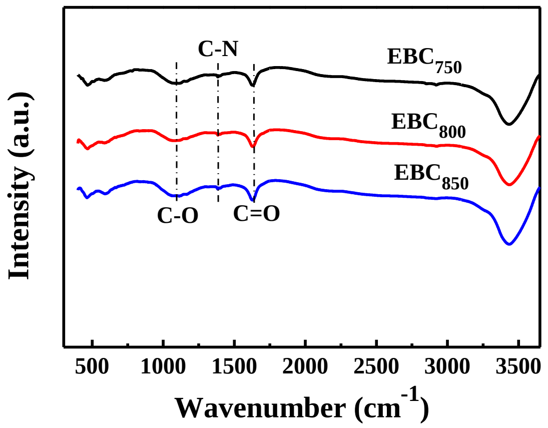

Fig. S3 FTIR spectra of EBC samples

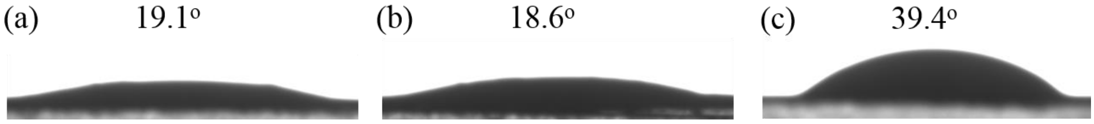

Fig. S4 Water contact angles of (**a**) EBC_{750}, (**b**) EBC_{800}, and (**c**) EBC_{850}

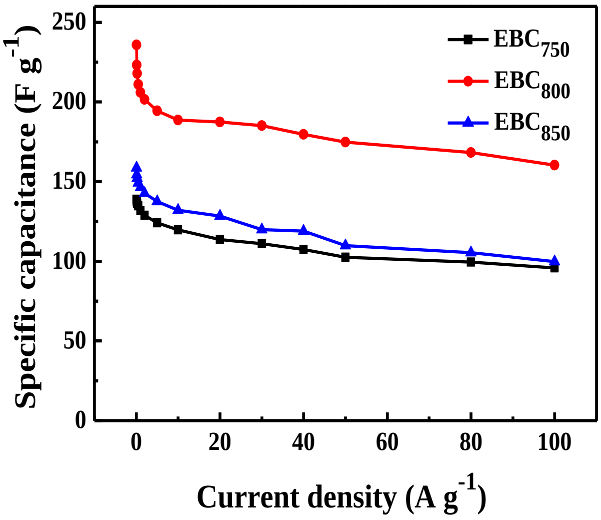

Fig. S5 Gravimetric capacitances of EBC electrodes at various current densities

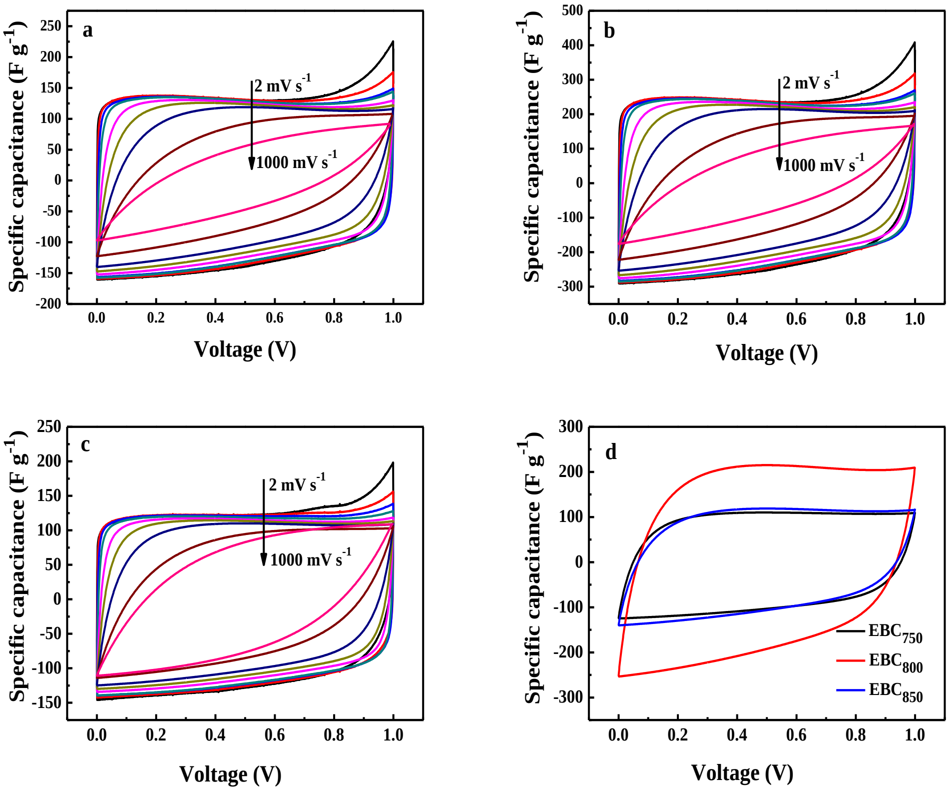

Fig. S6 CV curves of (**a**) EBC_{750}, (**b**) EBC_{800} and (**c**) EBC_{850} at various scan rates; (**d**) CV curves of EBC electrodes at the scan rate of 200 mV s^{-1}

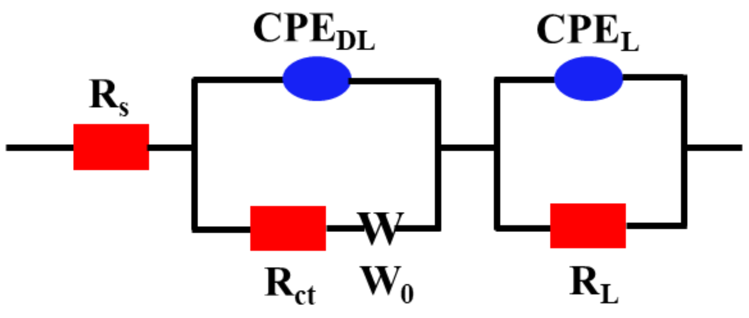

Fig. S7 Electric equivalent circuit model of EBC electrodes

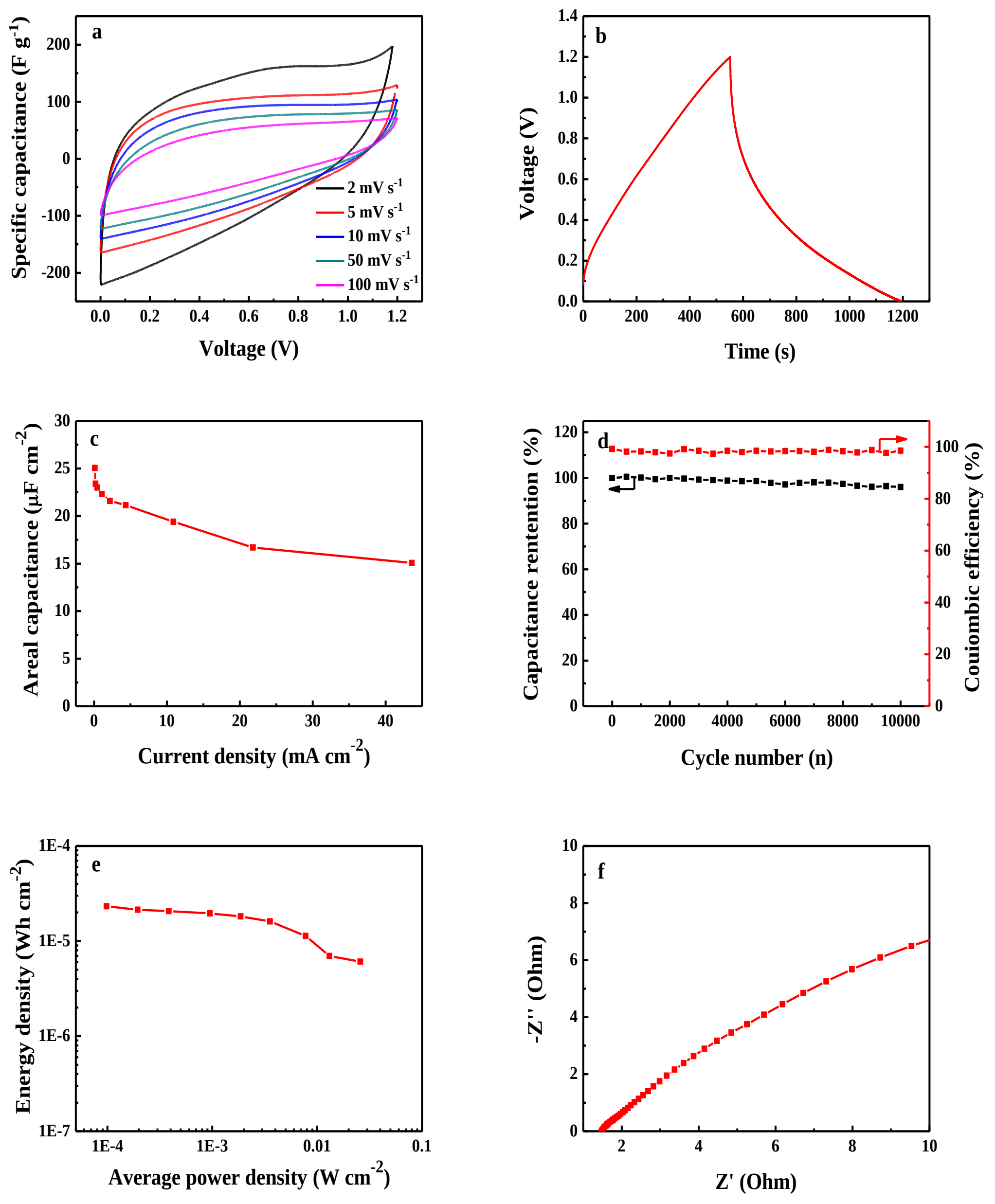

Fig. S8 The electrochemical performance of EBC_{800} electrode in the all-solid-state SC: (**a**) CV curves of EBC_{800} electrode at various scan rates; (**b**) GCD curve of EBC_{800}

electrode at the current density of 0.218 mA cm^{-2}; (**c**) areal capacitance of EBC_{800} electrode at various current densities; (**d**) capacitance retention and coulombic efficiency of EBC_{800}-based SC after 10,000 cycles at 10.9 mA cm^{-2}; (**e**) Ragone plots of EBC_{800}-based SC; (**f**) Nyquist plots of EBC_{800} electrod

Table S1 Crystallite lattice parameters of the EBC samples

Samples	d_{002} (nm)	L_c (nm)
EBC_{750}	0.3576	1.1574
EBC_{800}	0.3543	1.1737
EBC_{850}	0.3515	1.2417

Table S2 XPS results of the EBC samples

Samples	C 1s (at%)	O 1s (at%)	N 1s (at%)	O 1s (at%)		N 1s (at%)			
				C=O	C-O	N-6	N-5	N-Q	N-X
EBC_{750}	88.31	6.85	4.53	3.67	3.18	0.28	2.66	0.60	0.99
EBC_{800}	87.87	8.21	3.55	4.03	4.18	1.25	1.67	0.53	0.10
EBC_{850}	91.09	5.18	3.30	2.22	2.96	0.12	1.67	1.04	0.47

Table S3 Elemental analysis results of EBC samples

Samples	C (wt%)	H (wt.%)	N (wt%)	O (wt%)
EBC_{750}	88.88	0.73	4.15	6.24
EBC_{800}	87.66	0.80	3.41	8.13
EBC_{850}	90.34	0.68	3.12	5.86

S3 Supplementary References

[S1]G.N. Okolo, H.W.J.P. Neomagus, R.C. Everson, M.J. Roberts, J.R. Bunt, R. Sakurovs, J.P. Mathews. Chemical-structural properties of South African bituminous coals: Insights from wide angle XRD-carbon fraction analysis, ATR-FTIR, solid state ^{13}C NMR, and HRTEM techniques. Fuel **158**, 779-792 (2015). https//doi.org/10.1016/j.fuel.2015.06.027

[S2]R.C. Everson, G.N. Okolo, H.W.J.P. Neomagus, J.M.D. Santos, X-ray diffraction parameters and reaction rate modeling for gasification and combustion of chars derived from inertinite-rich coals. Fuel **109**, 148-156 (2013). https//doi.org/10.1016/j.fuel.2012.12.043
